# The impact of microbial keratitis on quality of life in Uganda

**DOI:** 10.1136/bmjophth-2019-000351

**Published:** 2019-12-23

**Authors:** Simon Arunga, Geoffrey Wiafe, Esmael Habtamu, John Onyango, Stephen Gichuhi, Astrid Leck, David Macleod, Victor Hu, Matthew Burton

**Affiliations:** 1 Department of Clinical Research, London School of Hygiene and Tropical Medicine, London, UK, UK; 2 Department of Ophthalmology, Mbarara University of Science and Technology, Mbarara, Uganda; 3 London School of Hygiene and Tropical Medicine International Centre for Eye Health, London, UK; 4 Carter Center, Addis Ababa, Ethiopia; 5 Department of Ophthalmology, University of Nairobi, Nairobi, Kenya; 6 Department of Clinical Research, London School of Hygiene & Tropical Medicine, London, UK; 7 ITD/CRU, LSHTM, London, UK; 8 Department of Medical Statistics, London School of Hygiene & Tropical Medicine, London, UK; 9 International Centre for Eye Health, London School of Hygiene and Tropical Medicine, London, UK; 10 Clinical Research Department, London School of Hygiene and Tropical Medicine, London, UK

**Keywords:** microbial keratitis, bacterial keratitis, fungal keratitis, blindness, quality of life, uganda

## Abstract

**Background:**

Microbial keratitis (MK) is a frequent cause of sight loss in sub-Saharan Africa. However, no studies have formally measured its impact on quality of life (QoL) in this context.

**Methods:**

As part of a nested case–control design for risk factors of MK, we recruited patients presenting with MK at two eye units in Southern Uganda between December 2016 and March 2018 and unaffected individuals, individually matched for sex, age and location. QoL was measured using WHO Health-Related and Vision-Related QoL tools (at presentation and 3 months after start of treatment in cases). Mean QoL scores for both groups were compared. Factors associated with QoL among the cases were analysed in a linear regression model.

**Results:**

215 case-controls pairs were enrolled. The presentation QoL scores for the cases ranged from 20 to 65 points. The lowest QoL was visual symptom domain; mean 20.7 (95% CI 18.8 to 22.7) and the highest was psychosocial domain; mean 65.6 (95% CI 62.5 to 68.8). At 3 months, QoL scores for the patients ranged from 80 to 90 points while scores for the controls ranged from 90 to 100. The mean QoL scores of the cases were lower than controls across all domains. Determinants of QoL among the cases at 3 months included visual acuity at 3 months and history of eye loss.

**Conclusion:**

MK severely reduces QoL in the acute phase. With treatment and healing, QoL subsequently improves. Despite this improvement, QoL of someone affected by MK (even with normal vision) remains lower than unaffected controls.

**Significance of the study**

**What is already known about this subject?**

- Quality of life (QoL) is affected in many bilateral ocular conditions such as cataract, glaucoma and trichiasis.
- The impact of microbial keratitis (MK) on QoL compared to unaffected individuals has not been previously reported.
- MK is a common cause of blindness in Sub-Saharan Africa.

**What are the new findings?**

- MK severely reduces QoL in the acute phase of the disease.
- QoL improves with treatment and healing.
- Despite improvement, QoL of someone affected by MK (even with normal vision) remains lower than unaffected controls.

**How might these results change the focus of research or clinical practice?**

- The focus of this study is to make the case that MK, although usually a uniocular disease severely reduces the QoL of the affected individuals.
- QoL is affected in many bilateral ocular conditions such as cataract, glaucoma and trichiasis.

## Background

Microbial keratitis (MK) has been described as a ‘silent epidemic’, which leads to substantial morbidity, related to sight loss, pain and stigma.^1^ It is the leading cause of unilateral blindness after cataract in tropical regions, estimated at 2 million cases of monocular blindness per year.^2 3^ 1.3 million individuals were bilaterally blind from corneal opacity globally (excluding trachoma and vitamin A deficiency), accounting for 3.2% of binocular blindness.^3^

Quality of life (QoL) is a very important consideration in the management of any disease and treatments should ultimately aim to maintain or restore QoL.^4^ Few studies from sub-Saharan Africa (SSA) have examined the effect of cataract and trachomatous trichiasis on QoL.^5–7^ These have generally examined both the general health-related quality of life (HRQoL) and more specific vision-related quality of life (VRQoL).

However, there is no published data on the impact of MK on QoL from SSA, and very little from other World regions. In the Mycotic Ulcer Treatment Trial 1 (MUTT1) in India which compared topical natamycin to topical voriconazole for the treatment of fungal keratitis, QoL scores at 3 months were compared between the two treatment arms. However, there was no comparison group of unaffected individuals.^8^ There is a need to better understand how MK and its outcomes affect people, to develop improved management, counselling and support.

To investigate the impact of MK on QoL, we conducted this study in South-Western Uganda. Here, we describe the QoL among patients with MK, at presentation and 3 months after presentation compared with the QoL of unaffected individuals recruited from the community who were individually matched for age, sex and location of residence.

## Methods

### Ethical statement

This study adhered to the tenets of the Declaration of Helsinki. It was approved by the London School of Hygiene & Tropical Medicine Ethics Committee (Ref 10647), Mbarara University Research Ethics Committee (Ref 10/04–16) and Uganda National Council for Science and Technology (Ref HS-2303). Written, informed consent in the local language was obtained before enrolment. If the potential participant was unable to read, the information was read to them, and they were asked to indicate their consent by application of their thumbprint, which was independently witnessed.

### Study design and participants

This study of the impact of MK on QoL was nested within a case-control study of MK in Uganda. We prospectively enrolled patients with MK that presented to Ruharo Eye Centre and Mbarara University and Referral Hospital Eye Centre from December 2016 to March 2018. These are the tertiary referral centres for South-Western Uganda. The case definition of MK was the presence of a corneal epithelial defect (of at least 1 mm diameter) with an underlying stromal infiltrate, associated with signs of inflammation (conjunctival hyperaemia, anterior chamber inflammatory cells, ±hypopyon).^9^ We excluded those not willing to participate or to return for follow-up, pregnant women, lactating mothers and those under 18 years. All the questions in the tools were responded to directly by the study participants.

### Assessment of cases

At presentation, we documented basic demographic information and ophthalmic history. Presenting Logarithm of Minimum Angle of Resolution visual acuity at 2 m in a dark room was measured using the Peek Acuity smartphone application.^10^ Cases were examined at a slit lamp to assess the anterior segment using a structured protocol. Corneal scrape specimens were collected from the ulcer at a slit lamp or an operating microscope and samples were processed in the department of microbiology laboratory at Mbarara University of Science and Technology. Following corneal scrapping, immediate Calcofluor White staining was done in the side laboratory at the eye hospital on a fluorescence microscope (Zeiss Primostar ILED) by the attending ophthalmologist to rule out fungal Keratitis. Additional microscopy (Gram staining and KOH staining) was done in the main University microbiology laboratory and results became available within 24 hours. Agar plates and broths (Blood, Chocolate, Potato Dextrose and Brain Heart) were incubated at 35°C–37°C for bacteria for up to 7 days and at 25°C for up to 21 days for fungi. Organism identification and sensitivity testing were performed using standard microbiological techniques. Cases were treated empirically at presentation and the treatment was reviewed when the microbiology results became available. Patients with fungal keratitis were treated with Natamycin 5% eyedrops (Zonat Sunways India), those with bacterial keratitis were treated with Ofloxacin 0.3% eyedrops (Biomedica Remedies-India). They were reviewed on days 2, 7, 21 and 90 (3 months). At 3 months, the cases were followed-up in their homes for a final assessment.

### Control recruitment

We recruited healthy community controls during the 3-month follow-up of cases in their home village. The controls were individuals without any current eye complaints and with normal vision. The controls were individually matched to the cases. They had to be living in same village as the case, be of the same gender and in the same age group (±5 years). Enrolment followed a similar approach to that previously used in study in Ethiopia.^6^ The research team visited the villages (typically 50–100 households), the local village head was asked to write down all individuals they thought would meet the matching criteria for a particular case in that village. One person was randomly selected from this list using a lottery method. They were approached and provided with details of the study and invited to participate if eligible. If a selected individual refused or was ineligible, another person was randomly selected and approached. Both cases and controls were asked about their social economic status, this was a self-reported question compared with their neighbours on a 5-scale level (5—‘very rich’, 4—‘rich’, 3—‘neither rich nor poor’, 2—‘poor’, 1—‘very poor’).^6 7 11^ In this 5-point scale, participants were asked ‘Compared with your neighbours, how do you rate your household wealth status?’

### QoL instruments

To measure QoL we used two instruments: the general health WHOQOL-BREF and the vision-related WHO/PBD-VF20. BREF stands for abbreviated form of the original WHOQOL tool. These were both initially independently translated by two translators into Runyankole, the local language. Any discrepancies were discussed with a third party and a merged final agreed version produced. Both instruments were administered to cases at presentation and at the 3-month follow-up. They were administered to the control group only once, during the 3-month assessment of the matched case.

#### VRQoL

the WHO/PBD-VF20 tool measures VRQoL. It assesses the impact of visual impairment in several domains including mental well-being, dependency and social functioning. The WHO/PBD-VF20 consists of 20 questions divided into four subscales: ‘General Vision’ subscale (one question); ‘Visual Symptoms’ subscale (three questions); ‘General Functioning’ subscale (12 questions); and ‘Psychosocial’ subscale (four questions). It begins by asking the patient ‘Overall, how would you rate your eyesight using both eyes?’; and uses a 5-point scale answer option such as ‘very good’, ‘good’, ‘moderate’, ‘bad’, ‘very bad’. Each subsequent question also has a 5-point response option: one indicates the highest and five the lowest score.

#### HRQoL

the WHOQOL-BREF (WHOQOL Group, 1998) has good applicability in low and middle-income countries as it was developed simultaneously from concept across 18 countries in Africa, Asia and Latin America.^12^ It measures four domains of health: Physical Health, Psychological Health, Social Relationships and Environment. It asks respondents 26 questions. These include the frequency they have experienced issues and/or were able to do things (eg, feel safe, able to concentrate, enjoy life) in the past 4 weeks and how satisfied they are with certain aspects of their lives (eg, sleep, capacity for work).^12^

### Sample size

Based on the effect sizes found in previous work on cataract and trichiasis, a sample size of 215 pairs would have 80% power to detect a moderate effect of MK on QoL with an effect size of 0.27 (effect size=QoL score difference (3)/SD 11) with a Type 1 error of 5%.^5 7^

### Analysis

Data were managed in Access (Microsoft), and transferred to Stata V.14 (StataCorp) for analysis. Data were analysed using a previously described methodology, applied in other QoL studies.^6 7 13^

#### VRQoL

All items were grouped, and scores added into their respective subscales: ‘General Vision’ subscale (one question); ‘Visual Symptoms’ subscale (three questions); ‘General Functioning’ subscale (12 questions); and ‘Psychosocial’ subscale (four questions). The subscale scores were then converted into a scaled value out of one hundred, using the formula: ((individual score—lowest possible score)/(highest possible score—lowest possible score)) × 100. Therefore, the person with the lowest possible VRQoL score would receive a scaled value of ‘0’ and the person with the highest possible VRQoL score receives a scaled value of ‘100’.

#### HRQoL

Data were analysed following the WHOQOL-BREF protocol.^12^ Three negatively framed items were reversed into a positive frame so higher scores denote higher QoL. To generate domain scores, questions were grouped into their respective domains and their scores totalled. The mean score of all items included in the domain was calculated and then multiplied by four. These scores then transformed to a 0 to 100 scale with the formula specified in the manual to allow comparison between domains made of unequal number of items.^14^

### Psychometric property evaluation

Construct validity of the VRQoL and HRQoL data was assessed through known-group difference and convergence validity using a linear regression model. Cronbach’s alpha was used to test for internal consistency and reliability of the VRQoL and HRQoL data.

Cases and controls were compared for baseline characteristics. However, we noticed that not all the pairs had been correctly matched for age because the village heads had subjectively guessed the ages of the controls. We thus adjusted for age throughout the analysis. The VRQoL and HRQoL analysis compared the cases to the controls at 3 months using a linear regression random effects model, which was adjusted for age and socioeconomic status, as these factors may confound the association between MK and QoL. A linear regression analysis was used to determine vision-related factors associated with QoL among the cases at 3 months adjusted for baseline QoL, age, sex, education and economic status.

### Patient and public involvement

Apart from helping to provide information during the piloting and data collection phase, we did not explicitly involve patients or the public in the designing and implementation of our work

## Results

A total of 313 MK cases presented and were enrolled. We were able to follow-up 260 cases at 3 months. It was not possible to enrol a control for 45/260 cases. Therefore, the analysis of QoL at 3 months comprises 215 pairs. The baseline characteristics were comparable among the cases and control group: median age was 47 years (IQR 35–60, total range 18–96 years), and 120 (56%) were male. (table 1)

**Table 1:** Baseline characteristics among the 215 case–control pairs (matched on gender and village and adjusted for age)

Exposure	Cases (215)	Controls (215)	P value
	n (%)	n (%)	
Married (yes) *	154 (72)	143 (67)	0.215
Head of household (yes) †	146 (68)	140 (65)	0.441
Education status ‡			
None	60 (28)	48 (22)	0.148
Primary	110 (51)	114 (53)	
Secondary	31 (14)	32 (15)	
Tertiary	14 (7)	21 (10)	
Farmer (yes) ‡	157 (73)	168 (78)	0.144
Size of the household §			
Small (1–4 people)	50 (23)	109 (51)	0.05
Medium (5–10 people)	115 (54)	94 (44)	
Large (>11 people)	50 (23)	12 (5)	
Self-reported wealth status ¶			
Poor	36 (17)	20 (9)	0.003
Middle	158 (73)	188 (88)	
Upper	21 (10)	6 (3)	

*People who were married, or cohabiting were considered as married while those who were divorced, single or widowed were considered as not married.

†Being head of the household meant people who were responsible for the overall care of the family, this was regardless of gender: among the cases and controls, 31% and 23% were female heads of households, respectively.

‡Majority of the participants had no or minimal education (primary level) which is not uncommon for a predominantly rural population in Uganda. Subsistence farming is the main occupation for this population.

§Majority of the household sizes were medium to large (five people or more). This is not uncommon since most of the living in rural Uganda is largely in an extended family setting.

¶Self-reported wealth status was classified as poor (1, ‘very poor’ and 2 ‘poor’), middle (3, ‘neither poor nor rich’) upper (4, ’rich’ and 5, ‘very rich’). There was one missing value among the control group. Participants were asked to compare themselves to their neighbours and give a score of their economic status.

Table 2 shows the baseline and 3-month VRQoL and HRQoL scores for the cases and the scores for the control group. The mean baseline VRQoL scores among the cases were all low (<50) except the psychosocial domain which had a score of 65 points. The most affected domain was visual symptoms, with a mean score of 20.7 (95% CI 18.8 to 22.7). The mean baseline HRQoL scores among the cases were all low (<50). At 3 months, all the case VRQoL and HRQoL scores had increased and were relatively high (between 80 and 91). Despite this increase, there was still very strong evidence (p<0.0001 in all domains) that QoL scores among MK cases at 3 months were lower than the controls, after adjusting for age, and economic status.

**Table 2:** VRQoL and general HRQoL among cases (baseline and 3 months) and controls (215 pairs)

Domain	Cases at baseline		Cases at 3 Months		Controls at 3 Months		Adjusted mean difference at 3 months		
	Mean	(95% CI)	Mean	(95% CI)	Mean	(95% CI)	Mean *	(95% CI)	P value †
VRQoL									
Overall sight	32.9	(30.6 to 35.2)	86.3	(83.3 to 89.2)	98.6	(97.5 to 99.6)	11.6	(8.8 to 14.5)	<0.0001
Visual symptom	20.7	(18.8 to 22.7)	88.3	(85.5 to 91.1)	99.4	(98.9 to 99.8)	10.5	(7.7 to 13.3)	<0.0001
General functioning	42.8	(40.6 to 45.1)	89.0	(86.1 to 91.9)	99.6	(99.3 to 100)	9.9	(7.1 to 12.7)	<0.0001
Psychosocial	65.6	(62.5 to 68.8)	90.7	(88.1 to 93.3)	99.8	(99.4 to 100)	8.5	(6.0 to 11.0)	<0.0001
HRQoL									
General facet items									
Overall quality of life	40.3	(39.0 to 41.6)	86.6	(83.9 to 89.4)	97.2	(96.4 to 97.9)	10.2	(7.6 to 12.8)	<0.0001
Overall Health	31.0	(29.0 to 33.0)	85.6	(82.7 to 88.5)	98.2	(97.3 to 99.1)	12.0	(9.1 to 14.9)	<0.0001
Domains									
Physical health	28.4	(26.5 to 30.3)	86.1	(83.1 to 89.2)	98.3	(97.6 to 98.9)	11.5	(8.6 to 14.5)	<0.0001
Psychological	49.2	(47.5 to 50.9)	84.4	(81.9 to 86.9)	94.0	(93.4 to 94.7)	9.1	(6.7 to 11.5)	<0.0001
Social	48.5	(46.5 to 50.5)	88.2	(85.2 to 91.2)	98.5	(97.6 to 99.3)	9.9	(6.9 to 12.9)	<0.0001
Environment	43	(41.7 to 44.3)	84.8	(82.0 to 87.6)	96.3	(95.2 to 97.3)	10.9	(8.2 to 13.6)	<0.0001

Only the cases who had controls were included in this analysis (215 pairs).

*Mean difference between cases and controls adjusted for age, sex and wealth status.

†Linear regression random effects model was used to test for significance of the differences among the cases and controls adjusted for age, sex and wealth status.

HRQoL, health-related quality of life; VRQoL, vision-related quality of life.

Table 3 shows the presenting vision, microbiology and 3-month outcomes among the 260 cases. Majority (137/260) presented with vision worse than 6/60 in the affected eye. Microbiology results were available for 226/260 participants out of which the majority (63%) showed fungal keratitis. At 3 months, 138/260 had vision of better than 6/18 in the affected eye. Vision had improved in 137 individuals, remained the same in 56 and worsened in 66 participants (sign rank p<0.0001).

**Table 3:** Presenting vision, microbiology and 3-month outcomes for the cases (n=260)

Variable	n/260 (%)
Presenting visual acuity in the affected eye (Snellen) *	
>6/18	86 (33)
6/18-6/60	36 (14)
<6/60	137 (53)
Presenting visual acuity in the non-affected eye (Snellen) *	
>6/18	232 (90)
6/18-6/60	14 (5)
<6/60	13 (5)
Microbiology †	
Fungal	143 (63)
Bacterial	18 (8)
Mixed	13 (6)
Unknown	52 (23)
Visual acuity in the affected eye (Snellen) at 3 months	
>6/18	138 (53)
6/18-6/60	37 (14)
<6/60	85 (33)
Visual acuity in the non-affected eye at 3 months	
>6/18	229 (90)
6/18-6/60	11 (4)
<6/60	15 (6)
Outcome at 3 months	
Healed no scar	34 (12)
Healed Mild scar	83 (30)
Healed moderate scar	65 (24)
Healed dense scar	46 (17)
Eviscerated	24 (9)
Not healed	20 (7)
Staphyloma	4 (1)

*There was one missing value (n=259).

†Corneal scrapping was performed on 226/260 participants; it was not possible to obtain corneal scrapping samples in 34 participants either due to uncooperative patient, declining consent, deep infiltrates with intact epithelium, such patients were treated based on clinical impression. In all, 52 samples returned negative, no organism detected on microscopy or culture, these were also managed based on clinical impression.

To investigate whether the difference in QoL between the cases and controls was due to factors in addition to impaired vision in the MK group, a separate subgroup analysis was performed comparing only MK cases with normal vision in the affected eye (better than 6/18) to their paired controls (online supplementary table 1). It was observed that the differences in QoL was similar to that obtained when using all the cases.

10.1136/bmjophth-2019-000351.supp1 Supplementary data

Tables 4 and 5 show factors associated with a good VRQoL and HRQoL among the cases at 3 months. This analysis was among all the 260 MK cases who were followed up at 3 months. Analysis was restricted to variables related to the disease such as vision at 3 months and whether the person had lost their eye. They were adjusted for sex, age, education, baseline QoL and socioeconomic status. Vision and eye loss were the both found to be associated with VRQoL and HRQoL.

**Table 4:** Univariable and multivariable linear regression for factors associated with VRQoL among cases only (n=260) seen at 3 months

Variable	Overall sight		Visual symptom		General functioning		Psychosocial	
	Mean	(95%CI)	Mean	(95%CI)	Mean	(95%CI)	Mean	(95%CI)
Visual acuity at 3 months								
>6/18	89.7	(86.6 to 92.7)	90.9	(87.6 to 94.2)	93.3	(90.5 to 96.1)	93.3	(90.9 to 96.1)
6/18–6/60	78.4	(69.1 to 87.6)	82.7	(73.5 to 91.8)	82.2	(73.3 to 91.1)	83.3	(74.9 to 91.7)
<6/60	77.4	(71.6 to 83.1)	82.9	(78.3 to 87.6)	81.3	(76 to 86.6)	83.8	(78.7 to 88.9)
P value*†	<0.0001		0.006		<0.0001		<0.0001	
P value‡†	0.001		0.044		0.006		0.003	
Eye removal §								
No	83.6	(80.7 to 86.6)	86.5	(83.5 to 89.3)	87.5	(84.6 to 90.3)	88.6	(86 to 91.2)
Yes	87.5	(76.3 to 98.7)	93.1	(86.3 to 99.8)	90.8	(81.6 to 100)	90.8	(81.2 to 100)
P value*	0.406		0.168		0.356		0.619	
P value**‡**	0.030		0.025		0.054		0.111	

*P values from univariable linear regression analysis.

†For visual acuity at 3 months (ordinal exposures with three categories), the p values were calculated for trend.

‡P values from multivariable linear regression analysis, adjusted for age, education status, wealth category and baseline QoL.

§Eye removal was a priori.

VRQoL, vision-related quality of life.

**Table 5:** Univariable and multivariable linear regression for factors associated with HRQoL among cases only (n=260) seen at 3 months

Variable	Overall QoL		Overall health		Physical health		Psychological		Social		Environment	
	Mean	(95% CI)	Mean	(95% CI)	Mean	(95% CI)	Mean	(95% CI)	Mean	(95% CI)	Mean	(95% CI)
Vision outcome at 3 months												
>6/18	88.7	(85.5 to 91.9)	88.8	(85.6 to 91.6)	89.4	(86.2 to 92.5)	87.7	(85.1 to 90.3)	90.6	(87.4 to 93.7)	87.9	(84.9 to 90.9)
6/18–6/60	82.1	(73.7 to 90.5)	82.0	(74.2 to 90.0)	81.9	(73.6 to 90.0)	80.9	(74.3 to 87.5)	84.8	(76.8 to 92.8)	78.8	(70.6 to 87)
<6/60	78.4	(73.3 to 83.3)	79.3	(74.0 to 84.5)	77.7	(72.0 to 83.5)	77.5	(72.9 to 82.1)	80.5	(74.6 to 86.3)	77.1	(71.9 to 82.3)
P value*†	<0.0001		0.001		<0.0001			<0.0001	0.002		<0.0001	
P value **‡†**	0.008		0.011		0.007			0.003	0.015		0.013	
Eye removal **§**												
No	84.0	(81.2 to 86.9)	84.5	(81.8 to 87.1)	84.0	(81.1 to 87)	83.0	(80.6 to 85.4)	86.4	(83.6 to 89.3)	82.5	(79.4 to 85.3)
Yes	88.0	(79.2 to 96.9)	88.1	(79.2 to 96.9)	89.1	(78.9 to 99.4)	86.8	(78.9 to 94.7)	87.8	(77.8 to 98.1)	88.2	(79.7 to 96.6)
P value*	0.356		0.402		0.288			0.331	0.633		0.198	
P value**‡**	0.038		0.031		0.028			0.041	0.122		0.009	

*P values from univariable linear regression analysis.

†For visual acuity at 3 months (ordinal exposures with three categories), the p values were calculated for trend.

‡P values from multivariable linear regression analysis, adjusted for age, sex, education status, wealth category and baseline QoL.

§Eye removal was a priori.

HRQoL, health-related quality of life.

Validity of the data was found to be good. Satisfying the known-groups difference criteria, the cases had significantly lower VRQoL and HRQoL scores in all domains (p<0.0001) than the controls (table 2). The VRQoL data were reliable after being assessed for internal consistency with a Cronbach’s alpha: coefficients of >0.80 (visual symptom 0.90, general functioning 0.98, psychosocial 0.87). The overall HRQoL data had a Cronbach’s alpha of 0.98 (physical health 0.96, psychological 0.89, social 0.91 and environment 0.95).

## Discussion

This study investigated the impact of MK on QoL in Uganda. Overall, both the VRQoL and HRQoL among MK patients at baseline was substantially reduced. The lowest scores were in the visual symptom (most had reduced vision) and physical health (most were in pain) categories. The least affected domain at baseline was psychological, which assesses ability to attend functions, feeling ashamed, feeling like a burden to others and fear of losing the other eye. In the only other published QoL study in people with MK, the MUTT1 study from India, QoL at the time of presentation was not reported.^8^

The QoL scores among the MK cases had improved greatly by 3 months. However, compared with the control group, there was strong evidence that MK results in a persistent reduction in QoL, with a mean difference in QoL scores of around 10 points. This effect remained evident even when MK cases with impaired vision were excluded from the analysis.

In looking at factors associated with QoL among MK cases at 3 months, after adjusting for other factors that may affect QoL, such as age, sex, education and economic status, visual acuity was an important determinant for both VRQoL and HRQoL. There was evidence that a history of eye loss was also associated with both VRQoL and HRQoL at 3 months. It was surprising to note that the people who had undergone evisceration had generally better QoL scores compared with those who did not. At the time of eye removal, most of the eyes were too damaged and painful that the people were ‘demanding’ for eye removal. They received socket prostheses (artificial eyes) after their eye removal procedures, this could have led to a marked reduction in pain and other unpleasant symptoms after evisceration, which led to a less impaired QoL compared with others who were not eviscerated where there would have been some with ongoing pain and other symptoms like dense corneal scars.

### Validity and reliability of QoL data

These tools have been used in a number of other vision-related studies to show a difference in QoL and have been reported to be valid and reliable in studies conducted in similar settings.^5–7^ Although the WHO/PBD-VF20 tool was designed to assess binocular vision, it has been demonstrated to be effective in detecting differences in monocular visual impairment in the MUTT1 trial where patients randomised to natamycin had a better 3-month visual-related QoL outcome compared with patients randomised to variconazole.^8^ In this study, both the VRQoL and HRQoL data measured what they were intended to measure (construct validity) by demonstrating evidence of differences in the scores between groups known to be different; MK cases and healthy controls had lower and higher scores, respectively. The VRQoL data also showed that subscales correlate well with measures of impact of MK on QoL such as visual acuity where worsening in these measures is associated with lower VRQoL subscale scores (construct validity). There was evidence of higher homogeneity among the items in each VRQoL and HRQoL subscale (internal consistency) than the generally accepted criteria of >0.70. Cronbach’s alpha scores ranged from 0.80 to 0.99 across all sub scales.

### Strengths/limitations

This was the first study to look at impact of MK on QoL and compare these with unaffected controls. The control selection was good and matched for age, sex and village. We used validated tools which have been previously applied in similar settings and the sample size was adequate to test for differences in QoL. This study did not collect baseline QoL information from the control group because it was not logistically possible. Information on other ocular comorbidities such as presence of a cataract and posterior segment disease at 3 months was not collected as these examination were conducted at patients’ homes and it was not practical to perform a full eye examination in such settings.

## Conclusion

This study showed that MK severely affects QoL in the acute phase. With treatment, QoL improves, with the highest QoL in cases who had little or no visual impairment at 3 months. Despite this impressive improvement, the QoL at 3 months of someone previously affected by MK (even when they have normal vision) remains lower than controls.
