# Supplementary material for: The impact of microbial keratitis on quality of life in Uganda
Source: BMJ Open Ophthalmol. 2019 Dec 23;4(1):e000351. doi: 10.1136/bmjophth-2019-000351 (PMC6936408; doi:10.1136/bmjophth-2019-000351)
Supplement: Supplementary data [file bmjophth-2019-000351supp001.pdf]

Supplementary Table1: 3 months Vision-Related and Health-Related Quality of Life scores among cases and controls where the cases had normal vision (>6/18), n=120 pairs

Domain	Cases		Controls		Adjusted mean difference		
	**Mean**	**(95%CI)**	**Mean**	**(95%CI)**	**Mean**	**(95%CI)**	**(P value)**
**VRQoL**							
Overall Sight	89.8	(86.5-93.2)	99.1	(98.1-100)	8.8	(5.4-12.3)	<0.0001
Visual Symptom	90.8	(87.2-94.4)	99.7	(99.4-100)	8.4	(4.9-12.0)	<0.0001
General Functioning	93.4	(90.4-96.4)	99.9	(99.7-100)	6.0	(3.0-8.9)	<0.0001
Psychosocial	94.2	(91.6-96.8)	99.8	(99.4-100)	5.3	(2.7-7.9)	<0.0001
**HRQoL**							
**General facet items**							
Overall quality of life	89.2	(86.1-92.3)	97.8	(97.1-98.5)	8.1	(5.0-11.2)	<0.0001
Overall Health	88.8	(85.3-92.2)	98.5	(97.2-99.7)	9.1	(5.5-12.7)	<0.0001
**Domains**							
Physical health	89.5	(86.1-92.9)	98.8	(98.0-99.6)	8.9	(5.4-12.3)	<0.0001
Psychological	87.7	(84.8-90.5)	94.6	(93.9-95.2)	6.6	(3.7-9.5)	<0.0001
Social	91.1	(87.7-94.5)	99.1	(98.2-99.9)	7.6	(4.1-11.1)	<0.0001
Environment	88.6	(85.5-91.7)	97.2	(96.0-98.4)	8.4	(5.2-11.6)	<0.0001

As part of the eligibility criteria, all controls had normal vision. However, in this analysis, only the control pairs where the cases had normal vision were analysed.
